# Water Absorption Kinetics in Composites Degraded by the Radiation Technique

**DOI:** 10.3390/ma14164659

**Published:** 2021-08-18

**Authors:** Elena Manaila, Gabriela Craciun, Daniel Ighigeanu, Maria Daniela Stelescu

**Affiliations:** 1 Electron Accelerators Laboratory, National Institute for Laser, Plasma and Radiation Physics, 409 Atomistilor St., 077125 Magurele, Romania; elena.manaila@inflpr.ro (E.M.); daniel.ighigeanu@inflpr.ro (D.I.); 2 National R&D Institute for Textile and Leather—Leather and Footwear Research Institute, 93 Ion Minulescu St, 031215 Bucharest, Romania; dmstelescu@yahoo.com

**Keywords:** natural rubber/plasticized starch composites, electron beam, degradation, water diffusion, kinetic

## Abstract

Rubber-based wastes represent challenges facing the global community. Human health protection and preservation of environmental quality are strong reasons to find more efficient methods to induce degradation of latex/rubber products in order to replace devulcanization, incineration, or simply storage, and electron beam irradiation is a promising method that can be can be taken into account. Polymeric composites based on natural rubber and plasticized starch in amounts of 10 to 50 phr, obtained by benzoyl peroxide cross-linking, were subjected to 5.5 MeV electron beam irradiation in order to induce degradation, in the dose range of 150 to 450 kGy. A qualitative study was conducted on the kinetics of water absorption in these composites in order to appreciate their degradation degree. The percentages of equilibrium sorption and mass loss after equilibrium sorption were found to be dependent on irradiation dose and amount of plasticized starch. The mechanism of water transport in composites was studied not only through the specific absorption and diffusion parameters but also by the evaluation of the diffusion, intrinsic diffusion, permeation, and absorption coefficients.

## 1. Introduction

The chemical structure of rubber can be changed in the production process through vulcanization, one of the desired effects being an increase in elasticity. The reverse process during which the formed bonds can be break down is so called devulcanization, rubber being transformed into an entirely new product. One of the goals of devulcanization is rubber recycling, a difficult process that is still made by high heat and toxic chemical usage [1,2,3].

Rubber degradation by ionizing radiation is fast, low cost, and environmentally friendly, producing active species such as free radicals, ions, and molecules, which can significantly modify the molecular structure of the irradiated material. The molecular modifications of organic polymers induced by ionizing radiation lead to chain branching and cross-linking, causing the increase of molecular weight of the polymer, but also chain scission or chemical degradation. These processes result in the breakage of the main chains of the macromolecule and a decrease of the molecular weight [4,5].

In this paper was investigated the kinetics of water absorption after degradation by irradiation with electron beams of elastomeric composites based on natural rubber (NR) cross-linked with peroxide in the presence of trimethylolpropane trimethacrylate (TMPT) and plasticized starch (PS). As a renewable, biodegradable, inexpensive, and widely obtainable biopolymer, the application of starch after plasticization is attractive due to obtaining of improved composite properties and an increased degradability degree [6].

The tested composites based on natural rubber and plasticized starch NR/PS were obtained by vulcanization with benzoyl peroxide. Generally, the structure of the elastomers thus vulcanized is characterized by C–C (due to NR cross-linking) and C–O links (due to the grafting of PS on the NR matrix). Theoretically, the carbon bonds (C–C) are penetrated with difficulty by solvent molecules due to strong bonding and high rigidity [7,8]. Water absorption depends on the degree of cross-linking, and the adhesion between filler and matrix largely determines the sorption behavior of the composite [9]. In the case of the composite materials based on elastomers and natural polymers, such as NR and PS, water absorption largely depends on hygroscopic or water-soluble components incorporated in the NR matrix, which behave as a semi-permeable membrane [9]. Due to the strong hydrophilic nature of the starch and the large interface area between the starch and the natural rubber matrix, the water absorption of the composite is expected to be strongly dependent on the amount of plasticized starch that has been used to obtain the composite.

The disruption of the C–C bonds in NR mainly leads to the cleavage of the macromolecular chain, and the disruption of the C–O bonds in NR/PS leads to the cleavage of the grafting bonds [10]. In terms of energy binding values, C–C and C–O bonds are comparable (346 vs. 358 kJ/mol), and it can be said that both phenomena occur equally in the irradiation process, which lead to the degradation of composite materials. In addition, the free radicals produced by irradiation in composites may contribute to the oxidation of functional groups, and the oxidized macromolecules can suffer chain cleavage with decreases in molecular weight [11]. For example, the effect of an accelerated electron beam on starch consists of glycosidic bond decomposition, which further leads to macromolecule decomposition and the appearance of molecules with smaller chains [12,13,14,15].

The aim of the paper was to conduct a qualitative study on the kinetics of water absorption in composites based on natural rubber and plasticized starch, obtained by benzoyl peroxide vulcanization and irradiated with electron beam, in order to appreciate their degradation degree. The starch content in composites and irradiation dose were the variables taken into account, and water absorption parameters (*Qt* and *Qeq*), diffusion parameters (*k* and *n*), diffusion coefficient (*D*), intrinsic diffusion coefficient (D*), absorption coefficient (*S*), and permeation coefficient (*P*) were the parameters and coefficients that were followed.

## 2. Materials and Methods

### 2.1. Materials and Sample Preparation

The properties and the quantities of rough materials and the methods for NR/PS composites obtained were conducted as described in our previous works [6,16,17]. Five types of composites, named here C0, C10, C20, C30, C40, and C50, as a function of PS loading, were obtained by dibenzoyl peroxide cross-linking in the presence of TMPT. These composites were subsequently subjected to electron beam (EB) irradiation of 5.5 MeV in atmospheric conditions and at the ambient temperature of 25 °C. In order to induce degradation, an ALID-7 linear electron accelerator built at the National Institute for Laser, Plasma, and Radiation Physics (Bucharest, Romania) was used. The irradiation doses were 150, 300, and 450 kGy. Samples were not covered with polyethylene foil, the presence of oxygen during irradiation being desired. The irradiation facility and method are described in our previous works [16,17,18,19].

There are many situations, usually correlated with sample contents but also with the irradiation dose and dose rate, when the temperature during irradiation increases over 150 °C. In order to avoid sample degradation due to the thermal phenomenon, we decided to stop the irradiation after every 50 kGy and measure the temperature. The results were as follows: C0—53.41 °C ± 0.1%, C10—48.41 °C ± 0.1%, C20—52.74 °C ± 0.1%, C30—54.64 °C ± 0.1%, C40—63.13 °C ± 0.1%, and C50—65.82 °C ± 0.1%. The samples were let to cool to room temperature of 25 °C between two irradiation of 50 kGys.

### 2.2. Laboratory Tests

#### 2.2.1. Water Absorption Tests and Measurements

In order to investigate the NR/PS composites degradation induced by EB irradiation, a qualitative study on the kinetics of water absorption was done. The SR EN ISO 20344/2004 standard was used for tests performed at room temperature of 23 ± 2 °C on circular samples of 15 mm diameter and 2 mm thickness. Three tests were realized for every sample as follows: the initial weights were determined before being placed in the laboratory oven for 24 h at 80 degrees (these are the necessary conditions for samples to reach constant mass), and then each sample was immersed in distilled water and reweighed with a precision of 0.1 mg at regular intervals of time until there were no more weight gains due to water absorption. The water uptake was calculated as follows:

(1) $$\mathrm{Water}\;\mathrm{uptake}\;(\%)=\frac{W_{s}-W_{1}}{W_{1}}\times 100$$

where *W_s_* is the weight of the sample saturated with water, periodically determined, and *W*_1_ is the initial weight of the oven-dried sample [20].

#### 2.2.2. Scanning Electron Microscopy (SEM)

Sample morphology was investigated using a scanning electron microscope (FEI/Phillips, Hillsboro, OR, USA). Samples were placed on an aluminum mount, sputtered with gold palladium, and then scanned at an accelerating voltage up to 30 kV.

## 3. Results and Discussion

### 3.1. Water Uptake

**Figure 1:**
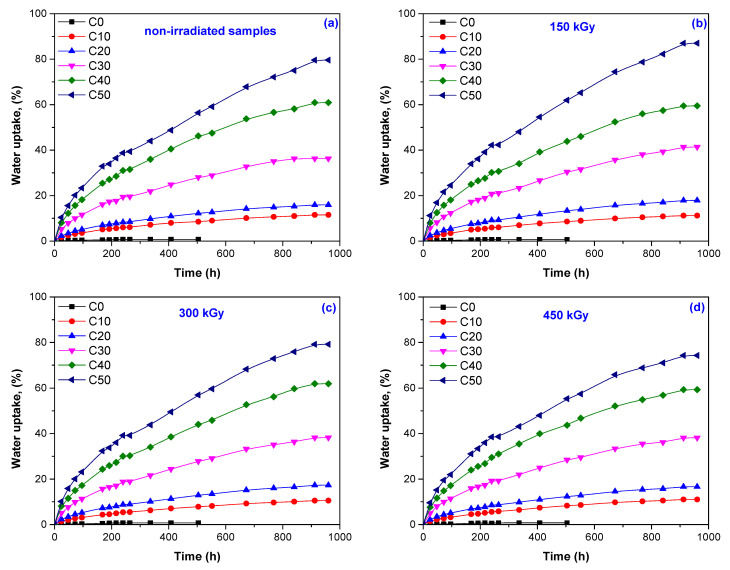
The water uptake (%) of non-irradiated samples—control (**a**) and irradiated at 150 kGy (**b**); 300 kGy (**c**); 450 kGy (**d**).

Water uptake tests were done on all C0–C50 samples, before and after irradiation, and the results are presented in Figure 1a–d and Table 1 and Table 2. The connection between water absorption, PS loading, and irradiation dose could be observed.

During the irradiation of polymeric materials, such as the NR/PS composites, both cross-linking and degradation can be induced as a function of irradiation dose and dose rate. More than that, both processes occur simultaneously but with different rates [21]. One of the effects of cross-linking is an increase of cross-linking degree, usually associated with low absorption percentages and mass losses after equilibrium has been reached. This can be observed by analyzing the results presented for samples C0 and C10 in Figure 1 and Table 1 and Table 2. The cross-linking process was dominant at the irradiation dose of 150 kGy. Here, the water uptake slowly decreased from 0.69% to 0.64% and from 11.52% to 11.20% in the cases of C0 and C10, respectively. Conversely, the weight loss slowly increased from 0.274% to 0.760% in the case of C0 and from 0.315% to 1.887% in the case of C10. The percentages of equilibrium sorption and mass loss at the equilibrium sorption were influenced by the irradiation dose (over 150 kGy) and PS amount, being quickly increased by these two. The increased PS amount, even in the non-irradiated samples, led to increased water uptake from 11.52% for the C10 sample to 79.60% for the C50 sample. Even when at 150 kGy a slowly increased water uptake was registered, once the irradiation dose increased to 300 and 450 kGy, the water uptake percentages decreased, a fact that can be associated with the increased cross-linking degree. However, correlating these results with those obtained for both water uptake and mass loss at equilibrium, it can be said that as the irradiation dose increased, the share of degradation reactions was higher than that of cross-linking. Thus, the water absorption in the tested composites was strongly influenced by the presence of water soluble and hygroscopic starch but also by the cross-linking degree and adhesion between PS and NR [9].

Generally, it is well known that elastomer cross-linking by means of peroxides follows that typical of a radical reaction. However, first the homolytic dissociation of peroxides in radicals occurs at the high temperature of 160 °C, followed then by the classical reaction of radical polymerization. Radicals formed react by addition to the double bond of natural rubber and produce a cross-linking reaction [22]. The bonds that are formed in the structure of elastomers that are vulcanized by means of peroxides are of the C–C type and are characterized by higher dissociation energy than those formed between macromolecular chains in the sulphur vulcanization systems. On the other hand, by PS grafting on the NR chain, bonds of the C–O–C type are formed [6,23].

Starch in the form of granules with variable sizes and shapes has a semi-crystalline structure and is insoluble in water (on contact with water, the starch granules swell). From a chemical point of view, two polysaccharide units are found in starch, namely amylose and amylopectin, both of which are composed of glucose units. The ratio between amylose and amylopectin is usually 30 to 70%, with only waxy starches containing more amylopectin [24,25,26,27,28,29]. Materials that contain starch are usually brittle, present many cracks on the surface, and are difficult to handle. However, all these disadvantages have been solved by the users through the addition of plasticizers to pure starch. In the present study, glycerin was used, the method being described in our previous works [6,30]. By plasticization, the mobility of polymeric chains, flexibility, and, as a consequence, the workability were enhanced. Additionally, the strong intermolecular interaction between the starch molecules was reduced [31,32,33]. The results presented in Figure 1 and Table 1 and Table 2, according to which the increase of the PS amount in composites and of irradiation dose led, at equilibrium, to decreased water uptake and increased mass loss, could be attributed to both plasticization and effects during irradiation. After EB irradiation of C10/C50 with 150/450 kGy, the water uptake decreased from 11.20% (C10/150 kGy) to 11.03% (C10/450 kGy) and from 87.01% (C50/150 kGy) to 74.27% (C50/450 kGy), and mass loss increased from 1.887% (C10/150 kGy) to 2.064% (C10/450 kGy) and from 3.335% (C50/150 kGy) to 4.009% (C50/450 kGy), respectively. A possible explanation for these results consists of the appearance of free radicals at the interaction between accelerated electrons and plasticized starch, which were able to induce molecular changes and fragmentation in starch molecule. Thus, by glycosidic linkage decomposition, the number of macromolecules having small chains increased, and smaller starch granules were formed [12,13,14,17]. Additionally, due to electron beam irradiation, both C–C and C–O scissions occurred. The C–C scissions in NR led to macromolecular chain scission, and C–O scissions in grafted PS led to grafting link scission [10,17]. FTIR spectra of NR/PS samples, irradiated with doses between 150 and 450 kGy, confirmed the oxidative degradation by irradiation (–CH_2_ deformation, –CH_3_ asymmetric deformation, –C=C– stretching vibration in the NR structure, OH stretching vibrations of absorbed water and carboxylate or conjugated ketone (>C=O)), and the results are presented in our previous work [17]. All resulting fragments were removed by immersion in water, and the results presented in Table 2 show this. The mass loss was due, on the other hand, to the increase of the starch solubility with the increase of the irradiation dose. In the presence of water, the starch swelling started in the amorphous regions. The amorphous phase swelling exerted tension on the crystallites in its vicinity, tending to distort them. The breakup of the amylopectin crystallite structure occurred due to the uncoiling or dissociation of double helical regions. The amylopectin released chains, hydrated, and swelled laterally, further disrupting the crystallite structure. The swelling power decreased with the irradiation increases due to amylose and amylopectin chain scission in starch molecules [34,35]. The decreased swelling power led to the increased starch solubility in water. Thus, the increase in irradiation dose was responsible for the increased starch solubility in water, and many researchers reported this connection as being associated with starch molecular degradation by irradiation [34,36,37,38].

### 3.2. Mechanism of Water Transport

Composite materials based on NR and PS are not completely soluble in water, but due to starch presence, they can absorb large quantities of water. Moreover, by applying the electron beam treatment for the purpose of degrading them, much information about the degradation process by damaging the matrix–fiber interface can be obtained [39]. Very useful are the diffusion tests made before and after irradiation. Water diffusion means the movement of water molecules in both the polymeric matrix and filler. The general mechanism of water molecule penetration in the composite material consists of capillary flow along the fiber–matrix interface followed by transport in micro-cracks. These mechanisms are active if the composite has suffered important changes and damages. The level of water molecule absorption may provide clues about the degradation process; the more degraded the composite material, the more water will be absorbed [39].

Some important aspects must be taken into account when the mechanisms of water transport are studied, namely the chemical structure of NR/PS, and the compatibility and adhesion between the rubber and filler [39]. Thus, in the degraded NR/PS, water will be found bonded through hydrogen bonds between rubber and filler and unbonded in the holes and nano-holes created in the degraded rubber [40].

The following equation was used in the present study in order to evaluate the mechanism of water transport in the NR/PS composite degraded by electron beam irradiation [39]:

(2) $$\log\frac{Q_{t}}{Q_{eq.}}=\log k+n\log t$$

where *Q_t_* (mol%) is the water uptake at time *t*, *Q_eq._* (mol%) is the water uptakes at equilibrium, and *k* and *n* are diffusion parameters. *k* and *n* are dependent on the nature of rubber and filler and on the adhesion at the interface between them.

*Q_t_* was calculated using the following equation [41]:

(3) $$Q_{t}=\frac{(W_{t}-W_{0})/M_{W}}{W_{0}}\times 100$$

where *W_t_* is the immersed sample weight for time *t*, *W*_0_ is the weight of dry samples, and *M_w_* is the water molar mass (18.0153 g/mol).

**Figure 2:**
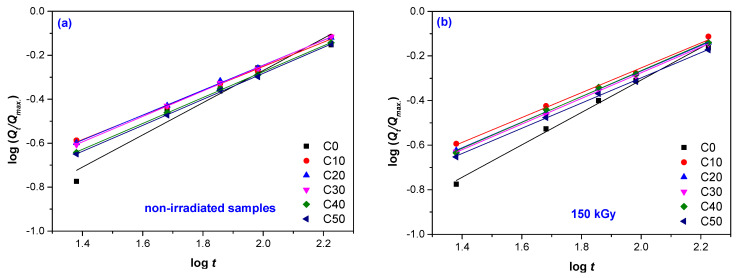

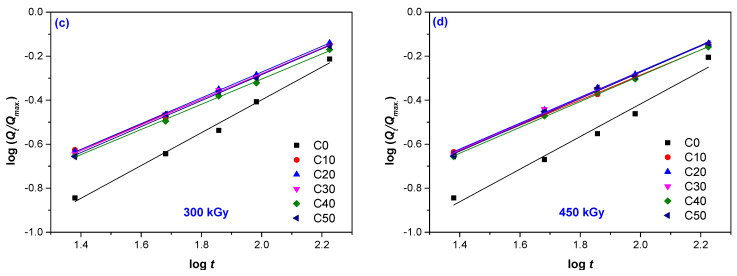
Plots of log(*Q_t_/Q_max._*) vs. log(*t)* used for diffusion parameters *n* and *k* for non-irradiated samples—control (**a**) and irradiated at 150 kGy (**b**); 300 kGy (**c**); 450 kGy (**d**).

The log(*Q_t_/Q_max._*) vs. log(*t*) plots for irradiated and non-irradiated samples are presented in Figure 2a–d and the obtained values for the diffusion parameters *k* and *n* in Table 3 and Table 4.

The diffusion parameters, *k* and *n*, were determined from the slope and y-intercept of the log (*Q_t_*/*Q_α_*) vs. log(*t*) plots. In the function of the values of *n* parameter, the type of water transport mechanism described by Fick’s theory is established [42]. Fickian diffusion (case I), associated with low water mobility comparative with the polymeric chain movement, has an associated value of 0.5 for the diffusion parameter *n*. In this case, the equilibrium is rapidly attained due to the lack of interaction between the rubber and filler. Anomalous diffusion has associated values of *n* between 0.5 and 1. Here, the water mobility is comparable with the movement of the polymeric chains. The non-Fickian diffusion (case II that corresponds to the value of *n* equal to 1 and super case II that corresponds to the value of *n* greater than 1) is associated with rapid water mobility comparable to polymeric chain relaxation [39,43].

The water transport mechanism in NR/PS composites depends on the fiber volume fraction, volume of voids, involved additives, humidity, temperature, orientation of reinforcement, fiber permeability, area exposed surface areas, diffusivity, reaction between water and rubber, and protection of the surface [40]. As seen in Table 3, samples without filler (C0) exhibited values of *n* between 0.727 and 0.746, which correspond to anomalous diffusion; water mobility is comparable to the weakening of polymer chain segments. EB irradiation led to an increase of *n* up to 0.746 for a 300 kGy irradiation dose. This corresponds to cleavage of the polymer chains in the network that is less dense and to a decrease of cross-linking degree. All these can be associated with the degradation process. The values of *n* for the samples containing PS (C10–C50) are still over 0.5 but are much closer compared to samples without PS. Moisture diffusion, desorption, or micro-crack formation are responsible for the deviation from Fickian behavior [39]. If the interaction between rubber and filler is strong, the amount of water absorbed is small, the situation being associated with low values of *n* [39]. In the case of non-irradiated samples C10–C50, the *n* value increased with increases in the PS content, a result that can be associated with a decrease of matrix–filler interaction. In the case of the same sample types, but irradiated, the *n* value increased also, displacing the transport of water in the anomalous area. The anomalous behavior is related to Fickian and non-Fickian transport mechanism coupling. Rubber segments have no time to respond to the stress generated by swelling and to rearrange themselves for future accommodation with the water molecules [44,45].

*k* is a constant of material that offers information about the interaction between the material structure and water. Low values of *k* correspond to a poor interaction between the material and water, the water absorption being also poor [46]. In Table 4 it can be seen, as was expected, that the smallest values of *k* were obtained for the samples without PS (C0). As the PS content increased, the values of *k* were higher but still decreased with increasing PS amount. By applying the EB treatment, *k* also decreased in all cases, C10–C50. Normally, high cross-linking degrees are associated with high values of the diffusion parameter *k* [46]. The decrease in *k* values with increasing irradiation dose shows that the cross-linking degree decreased, the composites degraded, and water easily penetrated the material.

### 3.3. Transport Coefficients

The transport coefficients are dependent on the solvent type and chemical composition of tested material. The water molecule is small compared to the macromolecules of NR/PS composites. The mechanism of water transport consists of water molecules penetration in the rubber/filler matrix followed by their diffusion in it. The difference between the number of molecules that have penetrated and those that have diffused is that which determines the overall water transport. After a certain time, the water absorption process strikes the equilibrium state that can be appreciated through the diffusion (D), intrinsic diffusion (D*), sorption (S), and permeation (P) coefficients [39].

**Figure 3:**
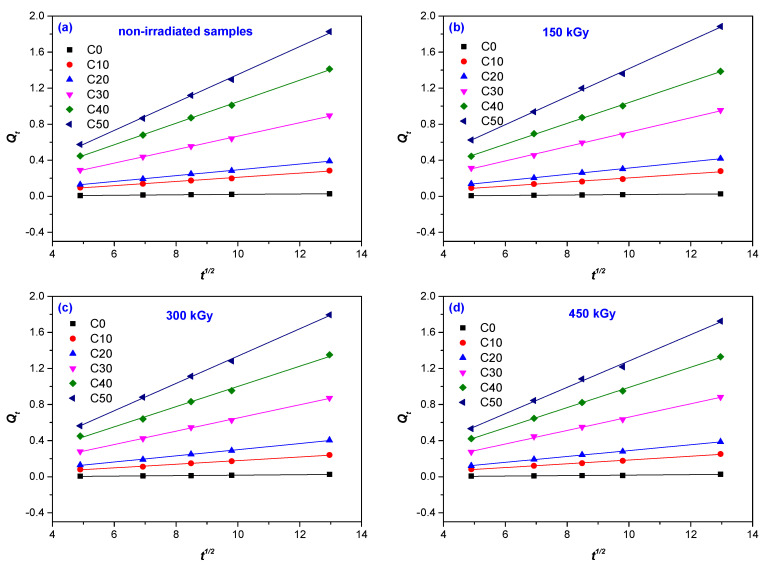
Plots of sorption data (*θ* is the slope of *Q_t_* vs. *t*^1/2^) of non-irradiated samples—control (**a**) and irradiated at 150 kGy (**b**); 300 kGy (**c**); 450 kGy (**d**).

The sorption curves *Q_t_* vs. *t*^1/2^ for the samples with and without PS are presented in Figure 3a–d. *D* was calculated from the slope *θ* of the initial linear portion, using the equation below [46,47,48]:

(4) $$D=\pi {\left(\frac{h\theta }{4Q_{eq.}}\right)}^{2}$$

where *h* is the thickness of the sample, and *Q_eq_*_._ (mol%) is the water uptake at equilibrium.

During the water sorption experiments, a significant swelling of NR/PS composites was observed, especially due to the relatively high PS content (between 10 and 50 phr). Thus, a correction was imposed of diffusion coefficients in the swollen state that was done by calculating D* from the volume fraction of material in the swollen sample, using the following equation [46,49]:

(5) $$D^{*}=\frac{D}{\Phi^{7/3}}$$

where *D* is the diffusion coefficient calculated with Equation (4), and Φ is the volume fraction of water, calculated as follows [46,50]:

(6) $$\Phi =\frac{W_{1}/\rho_{1}}{W_{1}/\rho_{1}+W_{2}/\rho_{2}}$$

where *W*_1_ is the weight of the composite sample and *ρ*_1_ is its density, and *W*_2_ is the weight of water and *ρ*_2_ is its density.

According to SR ISO 2781/2010, the composite densities were determined by the hydrostatic weighing method. The values of all types of NR/PS samples were found to be between 0.95 and 1.01 g/cm^3^. The NR density was 0.94 g/cm^3^.

The collective process of diffusion and sorption was appreciated by the permeation coefficient *P* that was calculated using the following equation [46,48]:

(7) $$P=D^{*}\times S$$

where D* and *S* are the intrinsic diffusion and sorption coefficients.

*S* is the sorption coefficient and it was calculated as below [46,51]:

(8) $$S=\frac{M_{eq.}}{M_{0}}$$

where *M_eq._* is the mass of water at the equilibrium swelling, and *M*_0_ is the initial mass.

Thermodynamic sorption constant at room temperature of 23 ± 2 °C, *Ks*, was calculated using the following equation [46,52]:

(9) $$K_{S}=\frac{\mathrm{No}.\;\mathrm{of}\;\mathrm{mols}\;\mathrm{of}\;\mathrm{solvent}\;\mathrm{sorbed}\;\mathrm{at}\;\mathrm{equilibrium}}{\mathrm{Mass}\;\mathrm{of}\;\mathrm{the}\;\mathrm{polymer}\;\mathrm{sample}}$$

The values of coefficients D*, *S*, *P*, and *K_S_* calculated as above are presented in Table 5, Table 6, Table 7 and Table 8.

Usually, a decrease of intrinsic diffusion coefficient, D*, is a consequence of increasing cross-linking degree [46]. As can be seen in Table 5, the intrinsic diffusion coefficient D* increased as the PS content also increased. The addition of PS in the composite matrix led to an increasing of availability of space for water molecules, which was even higher due to the hygroscopic properties of PS. This behavior showed a weak interaction between the polymeric matrix and filler that limits the water diffusivity in the composite [53]. By increasing the EB irradiation dose, D* of the C0 samples increased. This was associated with the decreasing of cross-linking degree due to the macromolecular chain cleavage reactions and degradation. For all C10–C50 samples, a slow decrease of D* with the irradiation dose increase was observed. These results, correlated with those obtained in mass loss experiments (Table 2), can be also attributed to the degradation process (cleavage of cross-linking or grafting links and PS solubilization) [10]. The same tendencies were observed for sorption coefficients (*S*), permeation coefficients (*P*), and thermodynamic sorption constants (*K_S_*). The results presented in Table 5, Table 6, Table 7 and Table 8 can be appreciated starting from the starch semi-crystalline structure (composed of the amorphous amylose and crystalline amylopectin) and its behavior in electron beam (EB) [24]. At room temperature, starch is insoluble. At 50 °C, starch may suffer gelatinization, pasting, and retrogradation [24,54]. Gelatinization is a two-stage endothermic process that affects both amorphous and crystalline areas. On the one hand, the breakage of hydrogen bonds in the amorphous portions happens. Thus, water manifests itself as a plasticizer, producing hydration and swelling in the amorphous regions. A decomposition of the intermolecular bonds in the starch takes place and thus more and more water is absorbed. On the other hand, the amylopectin structure is modified by EB irradiation, resulting in a reduction of crystallinity. Thus, the water absorption increases, causing several changes including solubilization and swelling [24]. The study of water absorption kinetics in composites based on NR and PS vulcanized with dibenzoyl peroxide is a promising indirect way to assess the degradation process induced by electron beam irradiation. Irradiation is an efficient method to induce modifications in the structure of NR/PS composites that are characterized by strong cross-linking and grafting bonds of the C–C and C–O type, which are very difficult to penetrate by water. C–C and C–O bonds are difficult to break and give the material a special rigidity, both characteristics being unfavorable to the water absorption process [7,8]. Irradiation in air as well as the appearance of specific favorable free radicals lead to the formation of oxidizing functional groups that contribute to the cleavage of macromolecular chains and the emergence of low molecular weight compounds that are either soluble in water or can be removed by immersion in water [11,12,13,14]. The results of the water absorption experiments as well as the study of the kinetics of the absorption phenomenon in the irradiated material demonstrate a difference between the irradiated and non-irradiated samples that can be attributed only to the degradation by irradiation.

### 3.4. Morphological Investigation by Scanning Electron Microscopy (SEM)

The results of tests that were made in order to evaluate the water uptake at equilibrium and mass loss after equilibrium sorption, as well as the modifications of specific diffusion parameters (*n*, *k*) and coefficients (*D*, D*, *S*, *P*, *K_S_*) after the electron beam irradiation, confirmed the appearance of modifications specific to degradation. Thus, the structural degradation by breaking the cross-linking and grafting bonds and the removal of some small molecule components formed after irradiation led to the appearance of cracks or holes in the composite material [39].

**Figure 4:**
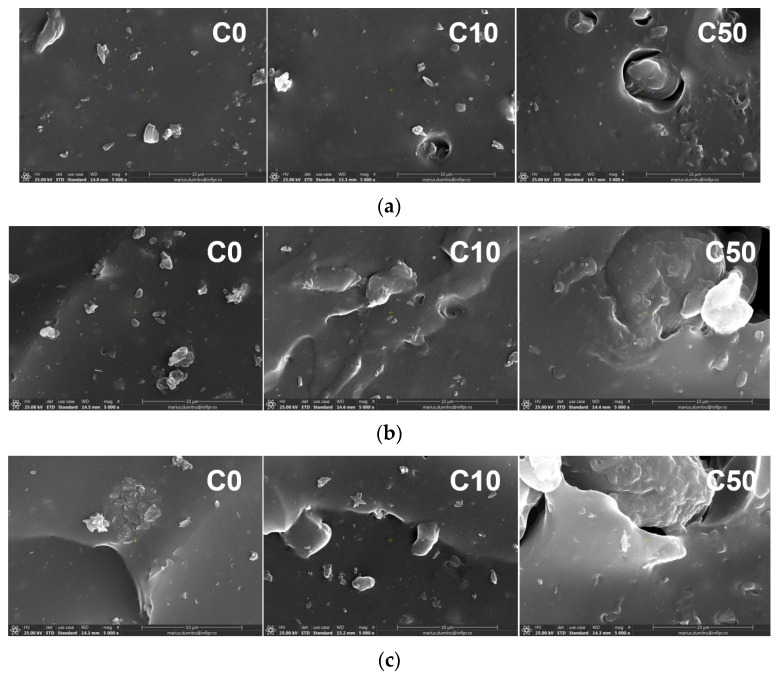

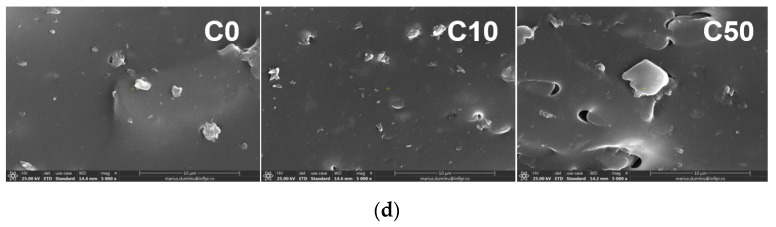
SEM micrographs of NR/PS samples vulcanized with dibenzoyl peroxide: non-irradiated C0, C10, and C50 samples (**a**); C0, C10, and C50 samples irradiated at 150 kGy (**b**); C0, C10, and C50 samples irradiated at 300 kGy (**c**); C0, C10, and C50 samples irradiated at 450 kGy (**d**).

The morphological changes of samples with and without PS were evaluated for comparison before and after irradiation by the SEM technique, and the resulting micrographs can be seen in Figure 4a–d. In the SEM micrographs, on the sample surfaces, small particles could be seen that could be either impurities that had not been removed by immersion in solvents or residues of vulcanization additives.

In Figure 4, it can be seen that non-significant changes on C0 sample surfaces were found. Surfaces were homogeneous and smooth, without holes or rough areas [55]. However, regarding the same samples irradiated at 300 kGy and 450 kGy, some cracks were found. On the SEM micrographs of non-irradiated C10 and C50 samples, some voids and PS particles non-adherent to the NR structure could be observed [56,57]. After irradiation, C10 and C50 samples showed visible surface changes due to the aging induced by oxidative degradation. As the irradiation dose increased on both C10 and C50 surfaces, some cracks and micro voids could be observed [55,58]. These suggest, on the one hand, a decreasing of the adherence at the interface between the elastomeric matrix and filler, and on the other hand, scissions of cross-linking and grafting bonds [59]. More cracks and micro voids were observed on the irradiated C50 sample surface, probably due to the increasing of filler loading [59].

All the morphological changes were specific to the degradation process and were sustained by the results obtained in the study of kinetics of water absorption being correlated with the PS content and irradiation dose.

## 4. Conclusions

Electron beam irradiation is a promising degradation method for replacing devulcanization, incineration, or simply storage and is usually used for rubber-based waste management. Polymeric composites based on natural rubber and plasticized starch in amounts of 10 to 50 phr and obtained by benzoyl peroxide cross-linking were subjected to irradiation in the dose range from 150 to 450 kGy using an electron beam of 5.5 MeV in order to induce degradation. Electron beam irradiation is the only non-polluting method that has the effect of breaking strong C–C and C–O type grafting/cross-linking bonds. Additionally, the appearance of oxidizing functional groups by irradiation in air contribute to macromolecular chains scission that reduces the molecular weight and makes the composites soluble or removable in water. Both C–C and C–O bonds, which give the material a special rigidity, are unfavorable to water absorption. In order to avoid sample degradation due to the thermal phenomena that can appear during irradiation (at every 50 kGy the sample temperature increased by 23 to 40 °C), the irradiations were performed by accumulating fragments of 50 kGy until the desired doses were obtained.

In order to assess the degradation process induced by irradiation, a study of water absorption kinetics in the composites was performed.

The values of *n* parameter showed that the water transport mechanism deviated from Fickian diffusion (*n* = 0.597 at the irradiation dose of 450 kGy for samples containing 50 phr of plasticized starch). The cleavage of the polymer chains in the polymeric network and the decreasing of cross-linking degree were attributed to moisture diffusion, desorption, or micro-crack formation being associated with deviations from Fickian diffusion. The intrinsic diffusion coefficient D* increased as the plasticized starch content also increased. The addition of plasticized starch in the composite matrix led to an increase of the availability of space for water molecules that was even higher due to the hygroscopic properties of PS. This behavior showed a weak interaction between the polymeric matrix and plasticized starch that limited the water diffusivity in the composite.

The results of the water absorption experiments as well as the study of the kinetics of the absorption phenomenon in the irradiated material demonstrated a difference in the irradiated samples compared to the non-irradiated ones that could be attributed only to the degradation by irradiation. The study of water absorption kinetics in composites based on natural rubber and plasticized starch vulcanized with dibenzoyl peroxide is an indirect but effective way to assess the degradation process induced by electron beam irradiation.

## Figures and Tables

**Table 1:** The percentages of equilibrium sorption (%).

Sample Codes	Filler Content (phr)	Equilibrium Sorption (%)			
		Non Irradiated	150 kGy	300 kGy	450 kGy
C0	0	0.69 ± 0.03	0.64 ± 0.21	0.71 ± 0.02	0.74 ± 0.05
C10	10	11.52 ± 0.47	11.20 ± 0.71	10.51 ± 0.38	11.03 ± 0.53
C20	20	15.92 ± 0.52	17.88 ± 0.22	17.31 ± 0.46	16.64 ± 1.07
C30	30	36.24 ± 0.86	41.34 ± 1.30	38.22 ± 1.88	38.17 ± 1.46
C40	40	60.89 ± 2.54	59.44 ± 5.02	61.93 ± 1.11	59.32 ± 2.94
C50	50	79.60 ± 3.35	87.01 ± 2.87	79.19 ± 3.69	74.27 ± 3.64

**Table 2:** The percentages of mass loss after equilibrium sorption (%).

Sample Codes	Filler Content (phr)	Mass Loss (%)			
		Non Irradiated	150 kGy	300 kGy	450 kGy
C0	0	0.274 ± 0.03	0.760 ± 0.21	0.753 ± 0.23	0.7440 ± 74
C10	10	0.315 ± 0.02	1.887 ± 0.04	2.058 ± 0.04	2.064 ± 0.21
C20	20	0.333 ± 0.01	2.347 ± 0.07	2.435 ± 0.04	2.480 ± 0.03
C30	30	1.115 ± 0.02	3.335 ± 0.11	3.626 ± 0.04	3.791 ± 0.04
C40	40	2.147 ± 0.05	3.339 ± 0.03	3.447 ± 0.11	3.370 ± 0.23
C50	50	3.087 ± 0.03	3.335 ± 0.23	3.355 ± 0.45	4.009 ± 0.43

**Table 3:** The values of *n* parameter.

Sample Codes	Filler Content (phr)	*n*			
		Non Irradiated	150 kGy	300 kGy	450 kGy
C0	0	0.733	0.727	0.746	0.741
C10	10	0.554	0.557	0.573	0.571
C20	20	0.568	0.573	0.585	0.590
C30	30	0.579	0.575	0.583	0.590
C40	40	0.589	0.851	0.572	0.587
C50	50	0.592	0.566	0.590	0.597

**Table 4:** The values of *k* (×10^−2^) parameter.

Sample Codes	Filler Content (phr)	*k* (×10^−2^)			
		Non Irradiated	150 kGy	300 kGy	450 kGy
C0	0	1.84	1.73	1.29	1.26
C10	10	4.37	4.28	3.75	3.74
C20	20	4.16	3.85	3.61	3.57
C30	30	3.93	3.75	3.58	3.53
C40	40	3.52	3.74	3.56	3.45
C50	50	3.42	3.72	3.43	3.39

**Table 5:** The values of intrinsic diffusion coefficient, D* (×10^−9^ m^2^s^−1^).

Sample Codes	Filler Content (phr)	D* (×10^−9^ m^2^s^−1^)			
		Non Irradiated	150 kGy	300 kGy	450 kGy
C0	0	3.257	2.729	3.195	3.346
C10	10	3.901	3.995	3.470	3.463
C20	20	4.244	4.387	4.313	4.314
C30	30	6.335	6.313	5.942	5.947
C40	40	9.145	8.870	7.521	7.436
C50	50	10.914	9.824	11.010	11.119

**Table 6:** The percentages of sorption coefficient, *S* (%).

Sample Codes	Filler Content (phr)	*S* (%)			
		Non Irradiated	150 kGy	300 kGy	450 kGy
C0	0	0.00672	0.00762	0.00721	0.00745
C10	10	0.112	0.115	0.109	0.110
C20	20	0.159	0.185	0.175	0.172
C30	30	0.362	0.413	0.393	0.382
C40	40	0.581	0.612	0.625	0.626
C50	50	0.796	0.916	0.808	0.743

**Table 7:** The values of permeation coefficient, *P* (×10^−10^ m^2^s^−1^).

Sample Codes	Filler Content (phr)	*P* (×10^−10^ m^2^ s^−1^)			
		Non Irradiated	150 kGy	300 kGy	450 kGy
C0	0	0.235	0.207	0.235	0.240
C10	10	4.486	4.564	3.697	3.724
C20	20	6.880	7.578	7.395	6.952
C30	30	23.38	25.48	22.31	22.67
C40	40	53.08	49.87	46.61	47.08
C50	50	88.68	90.01	85.02	79.21

**Table 8:** The values of thermodynamic sorption constant, *K_S_* (mol kg^−1^).

Sample Codes	Filler (phr)	Non Irradiated	*K_S_* (mol kg^−1^)		
			150 kGy	300 kGy	450 kGy
C0	0	0.362	0.341	0.376	0.392
C10	10	5.970	5.906	5.530	5.738
C20	20	8.377	9.398	9.115	8.747
C30	30	19.08	21.76	20.11	20.08
C40	40	30.35	32.18	33.22	32.24
C50	50	41.67	46.97	41.66	39.03

## Data Availability

The study did not report any data.
